# CT Utilisation in Emergency Department (ED) Assessment of Patients With Suspected Polytrauma: Impact of a Dedicated Trauma Surgical Team

**DOI:** 10.1111/1754-9485.13843

**Published:** 2025-02-22

**Authors:** Rebecca Hong, Salma Qassin, Chris Zhao, Nihal Raju, Zemar Vajuhudeen, Danielle Thom, Casey Paton, Leonid Churilov, Odkhishig Ganbold, Natalie Yang, Gerard Smith, Ruth P. Lim

**Affiliations:** ^1^ Department of Radiology Austin Health Heidelberg Victoria Australia; ^2^ Melbourne Medical School The University of Melbourne Parkville Victoria Australia; ^3^ Department of Radiology Melbourne Medical School Parkville Victoria Australia

**Keywords:** polytrauma, trauma surgical unit, WBCT

## Abstract

**Introduction:**

We aimed to assess the impact of introduction of a dedicated trauma surgical unit (TSU) on CT utilisation for polytrauma in the Emergency Department (ED).

**Methods:**

Single centre retrospective cohort study comparing adult patients undergoing CT for polytrauma following TSU introduction (Intervention group, *n* = 617) to a historical Baseline group (*n* = 257) over a matched time period. Patient impact, including initial clinical assessment, injuries, radiation exposure, incidental findings, ED disposition, and impact on radiology services were compared with Mann–Whitney and Fisher's exact tests.

**Results:**

Intervention patients were more likely to be examined by ED physicians (96.7% vs. 91.1%, *p* = 0.001) prior to CT. There was greater documented clinical suspicion for chest and abdominal injuries, with increased WBCT utilisation for Intervention (Baseline 17.1% vs. 47.8%, *p* < 0.05), with no significant increase in positive scans by region. More CT chest (Intervention 38.4% vs. Baseline 14.8%, *p* < 0.05), CT abdomen (42.6% vs. 12.6%, *p* < 0.005) and CT pelvis (46.1% vs. 16%, *p* < 0.001) was performed even with low documented clinical suspicion, with no significant increase in positive findings. The intervention group returned for more additional scans (12.48% vs. Baseline 5.45%), had more incidental findings (23.66% vs. 15.18%), and were more likely to be admitted for observation (21.7% vs. 14%), all *p* < 0.05. Time to scan and total CT reporting time were significantly longer for Intervention.

**Conclusion:**

Introduction of a TSU was associated in a shift towards increased CT utilisation, with no increase in scan yield, increased incidental findings and impacts on Radiology workflow.

## 1 Introduction

Trauma was the fourth most common presentation to Australian Emergency departments (ED) in 2022–23, and the most common presentation in patients aged 5–54 years and above age 85 [1]. Imaging is crucial in trauma assessment, with improved patient outcomes the closer the trauma room is to the computed tomography (CT) scanner [2]. Advanced Trauma Life Support (ATLS) guidelines recommend plain radiography and Focused Assessment with Sonography for Trauma (FAST) scans to guide immediate management, followed by selective CT imaging to regions of suspected injury [3]. However, with the speed and ready availability of CT, demand to meet ED standardised targets and improve hospital throughput, there has been a trend towards early referral for whole‐body computed tomography (WBCT) [4, 5, 6].

Potential patient advantages of WBCT include prompt diagnosis, less time spent in ED and increased sensitivity for occult injuries. While retrospective studies report a non‐significant absolute mortality reduction with initial WBCT (20.5% vs. 22.1%) [7, 8], an international multi‐centre randomised trial, REACT‐2, found no reduction in in‐hospital mortality [9].

However, widespread use of WBCT has potential negative consequences for patients and radiology services [6]. Gordic et al. found that WBCT for trauma led to a significantly higher effective dose exposure of 29.5 m‐sieverts (mSv) compared to 15.9 mSv for non‐WBCT [10]. Radiation over 20 mSv corresponds to an attributable cancer risk of greater than 1/1000 [11]. WBCT contributes to Radiology department workload, with downstream impacts on study turnaround time, radiology burnout and potentially reporting accuracy [12].

Dedicated trauma surgical units (TSU) specialise in delivering care to trauma patients from initial presentation to discharge and are associated with reduced mortality, length of stay and lower costs [13, 14]. A 20%–33% reduction in mortality has been reported in North American level 1–3 trauma centres [15, 16] and also for severely injured patients in an Australian Level 1 trauma centre [14]. Level 1 and 2 trauma centres offer comprehensive care to major trauma patients, while Level 3 and 4 centres focus on stabilisation before transferring patients to higher‐level centres [17]. While Ursic et al. briefly noted no difference in the costs from radiology imaging requests after the introduction of their TSU [14], no study has delved into the impact of such services on CT utilisation from the patient and Radiology department perspective in Australia.

A TSU, the General And Trauma Emergency Surgery Unit, was implemented at our Level 2 trauma service hospital in July 2021. This study aimed to retrospectively assess the impact on suspected polytrauma CT imaging pre‐ and post‐introduction of this TSU. We hypothesised its introduction would be associated with an increase in CT utilisation. We aimed to assess impact on patients, including injuries, disposition from the ED, radiation exposure and incidental findings; and radiology services, including initial CT scan coverage, scan and reporting turnaround times, as well as the use of additional CT.

## 2 Methods

### 2.1 Study Design

Retrospective cohort single‐centre study at a Level 2 trauma centre in Melbourne, Australia, with institutional ethics approval. Patients were identified via a search of the Radiology Information System (RIS, Impax v6.3.3, Agfa Healthcare, Mortsel, Belgium), for patients who underwent an ED referred CT cervical spine (CT‐C spine) scan plus any additional region scanned. All scans were performed on the same dual source multi‐detector CT (Siemens Force, 2 × 192 detectors) for ED patients with routinely obtained fine slice imaging in the trauma setting with bone reconstructions ≤ 1 mm (representative CT parameters presented in Table S1). Patients < 18 years old, presenting for isolated extremity or low‐impact head injury, or who underwent CT‐C spine for a non‐trauma related indication were excluded.

The intervention group (Intervention) were ED trauma patients who underwent CT for suspected polytrauma between October and December 2022, following institution of the TSU 15 months prior. A pre‐COVID‐19 time period baseline group (Baseline) in a matched time period (October to December 2019) was selected [18]. These time frames were also selected to avoid the extended periods of lockdown that impacted our city in 2020 and 2021 [19], when reduced trauma admissions were reported [18].

### 2.2 Data Collection

Clinical data collection was performed by four medical officers with one to 3 years of clinical experience, supervised by a radiologist with 18 years' experience, utilising the institution's electronic medical record (PowerChart v2018.01, Cerner Corporation, Kansas City, MO, USA). Radiation dose, time to scan and reporting times were extracted from the RIS.

### 2.3 Patients

#### 2.3.1 Baseline Characteristics

Age at presentation, sex, anticoagulant use, place of residence as a proxy of functional status (independent at home, home with carers or from a nursing home), and factors impairing communication, defined as a history of cognitive impairment (e.g., dementia), altered consciousness measured by reduced Glasgow Coma Score (GCS) < 14, intoxication or a non‐English speaking background were recorded.

#### 2.3.2 Presentation Characteristics

Triage heart rate (HR), systolic blood pressure (SBP), respiratory rate (RR), oxygen saturation (SpO_2_), GCS and presentation haemoglobin (Hb) were recorded. Initiation of a hospital‐wide or ED‐based trauma call was recorded and whether patients were considered to have a high‐risk mechanism of injury.

A hospital‐wide trauma call necessitating the presence of ED, surgical team and anaesthetics, is called for patients with either (i) significant vital sign derangements and/or (ii) severe injuries (e.g., suspected airway compromise, penetrating injuries, threatened limb). Patients with stable vital signs with uncomplicated or isolated injuries (e.g., isolated pelvic fracture) would typically meet criteria for an ED‐based trauma call which requires the presence of a senior ED physician. Patients with high‐risk mechanisms of injury alone (e.g., motor vehicle accident > 60 km/h without vital sign derangement or obvious significant injury) qualify for an urgent ED review instead of a trauma call. Patients who do not undergo a trauma call would subsequently be referred to the surgical trauma team after ED assessment.

Prior to establishing a TSU, all trauma referrals were received by the general surgical teams. Since implementation, during business hours all trauma cases are referred to the TSU, which is staffed by a consultant surgeon, two unaccredited registrars and two residents. After‐hours, trauma referrals are directed to the on‐call general surgical service, which included the TSU 1 in 4 times, regardless of whether TSU or another general surgical unit is receiving after hours. This is staffed by an unaccredited general surgical registrar (typically has at least 3 years of clinical experience) who manages referrals with consultant support. Overnight trauma patients are transferred to the TSU during working hours for continued management.

#### 2.3.3 Clinical Evaluation Prior to CT

Clinical examination before CT was recorded, including whether examination was performed by an ED physician or surgical team member, involvement of the receiving surgical team prior to CT scan (including telephone consultations), and documented clinical suspicion for injury by region (head, neck, chest, abdomen, pelvis and other regions, i.e. thoracolumbar spine).

#### 2.3.4 Initial CT

Prevalence and type of injury by region (head, cervical spine, chest, abdomen, pelvis, and other, e.g. targeted thoracolumbar spine) were recorded. Incidental findings were categorised by clinical significance (categories: 1 = inpatient workup required, e.g. aortic aneurysm > 5 cm; 2 = outpatient workup; 3 = no follow‐up required, e.g. simple renal cysts, degenerative lumbar spine disease etc. [20]), with all category 1 and 2 findings recorded. The proportion of patients with injuries by clinical suspicion and patient radiation dose (dose‐length product, DLP) from each presentation CT was also recorded.

#### 2.3.5 Patient Disposition From ED

Patient disposition was recorded as: discharged to baseline accommodation, transferred to short stay unit (for monitoring or further workup within 24 h), admission to ward or ICU, requiring emergency operation, or death in ED.

### 2.4 Radiology Impact

#### 2.4.1 CT Scan Coverage and Yield

CT coverage, including use of WBCT versus selective CT, was recorded. CT regions were divided into: head (CTB), cervical spine (CT‐C spine), chest (CT‐chest), abdomen (CT‐abdo), pelvis (CT‐pelvis) and other CTs (CT‐other). Utilisation of WBCT in patients with communication barriers was also assessed. CT scan yield (defined as one or more trauma‐related positive findings in a region divided by the total number of regions scanned) was assessed.

#### 2.4.2 Time to Scan and Additional Scans

Time from ED presentation to initial CT was recorded. Additional CT within 48 h of presentation for further trauma workup (“completion scans”) and the time to these scans were recorded.

#### 2.4.3 Reporting Time

Time to report the initial CT scan in minutes for trainees and consultants was recorded (time the report was actively open for reporting as recorded in the RIS). At our institution, some in‐hours and the majority after‐hours ED CT imaging is preliminarily interpreted by radiology trainees, with reports reviewed and finalised by a consultant radiologist, from almost immediately (in‐hours) to up to 18 h (after‐hours) after the preliminary report. Total interpretation time by number of regions scanned was also evaluated.

### 2.5 Statistical Analysis

Baseline and Intervention patient and imaging characteristics were compared using the Wilcoxon Mann–Whitney rank‐sum test for continuous variables and Fisher's exact test for categorical variables. We compared the odds of injury detection between groups using random‐effects logistic regression to account for repeated measurements (multiple scans) within individual patients. Statistical analysis was performed in Stata SE 18.0 (StataCorp Texas, USA), with a two‐tailed *p*‐value of < 0.05 considered statistically significant.

## 3 Results

### 3.1 Patient

**FIGURE 1:**
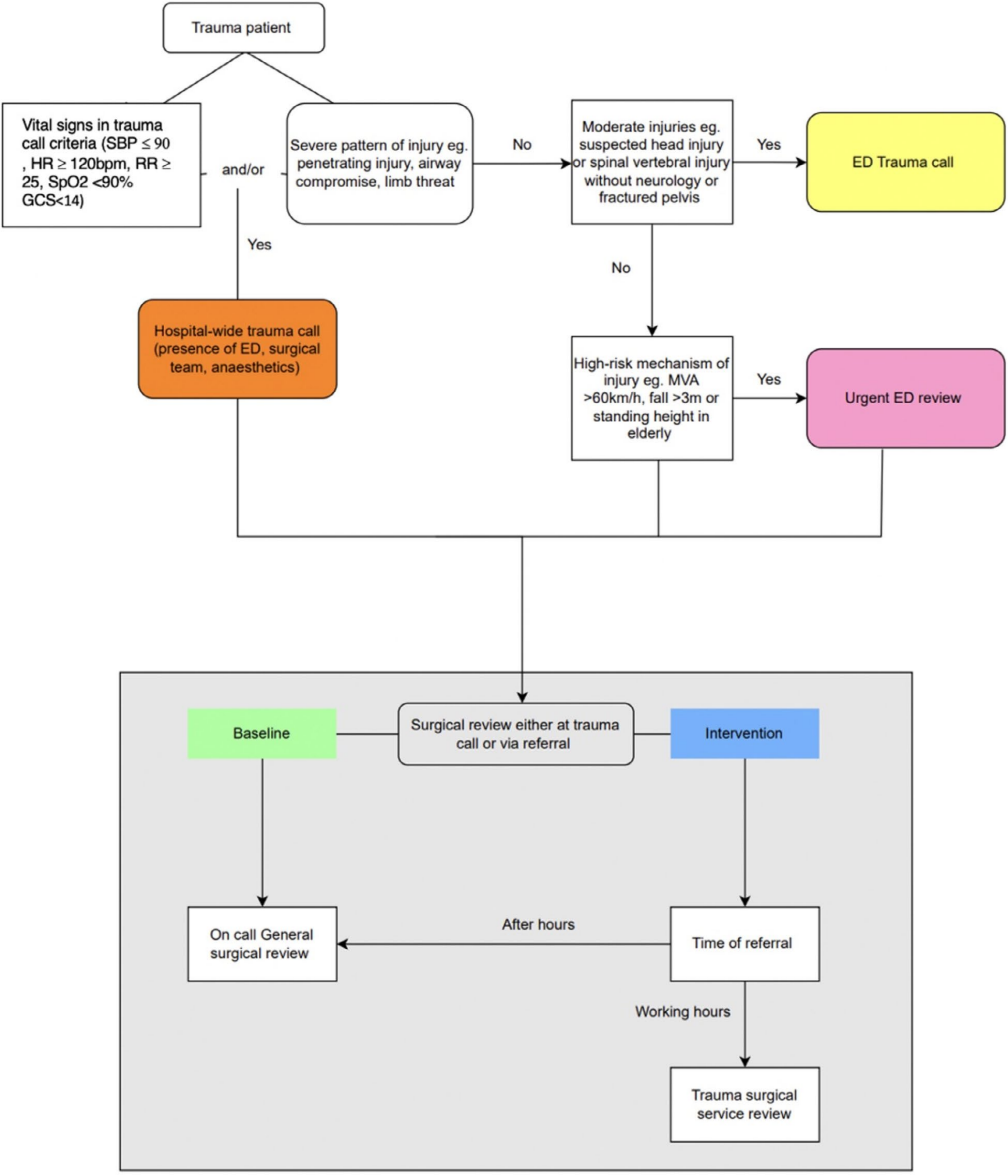
Trauma pathway at our level two institution demonstrating the pathway leading to a hospital‐based trauma call (orange), ED trauma call (yellow) and urgent ED review (pink). The lower half of figure (grey box) demonstrates the difference in surgical unit referrals before (baseline, green) and after the introduction of dedicated trauma surgical service (intervention group in blue). For after‐hours referrals in the Intervention group, the TSU was the on call unit 1 in 4.

There were 257 Baseline patients and 617 Intervention patients (Table 1). There was no statistically significant difference between groups in terms of age (69 ± 20.7 years vs. 70.9 ± 20.2 years, Baseline and Intervention respectively), gender (42.4% vs. 49.3% male), those on anticoagulation (36.2% vs. 35.5%) and patients with factors impairing communication (51% vs. 46.8%), *p* all > 0.05. Functional status as reflected by living situation was also similar between groups, with the majority living independently at home (75.1% vs. 70.5%) (Figure 1).

**TABLE 1:** Patient characteristics and clinical presentation.

	Baseline group	Intervention group	*p*
Number of patients	257	617	
Age (mean ± SD)	69 ± 20.7	70.9 ± 20.2	> 0.05
Male *n* (%)	109 (42.4%)	304 (49.3%)	0.074
Patients anticoagulated *n* (%)	93 (36.2%)	219 (35.5%)	0.877
Impaired communication *n* (%)^a^	131 (51.0%)	289 (46.8%)	0.266
Living situation			
Nursing home *n* (%)	38 (14.8%)	100 (16.3%)	0.323
Home with carers *n* (%)	26 (10.1%)	82 (13.3%)	
Independent at home *n* (%)	193 (75.1%)	433 (70.4%)	
HR median (IQR)	80 (20)	80 (24)	0.369
Tachycardia *n* (%)^b^	9 (3.5%)	24 (3.9%)	0.848
SBP (mean ± SD mmHg)	141 ± 27.4	137.1 ± 24.9	0.112
Hypotensive *n* (%)^b^	3 (1.2%)	10 (1.6%)	0.765
RR median (IQR)	17 (3)	18 (4)	< 0.001*
Tachypnoea *n* (%)^b^	10 (3.9%)	31 (5%)	0.599
SpO2 (mean ± SD %)	97 ± 2.6	96.1 ± 2.5	< 0.001*
SpO_2_ ≤ 90%^b^	4 (1.56%)	3 (0.49%)	0.204
GCS median (IQR)	15 (1)	15 (1)	0.719
GCS < 14^b^	19 (7.4%)	31 (5%)	0.200
Mean ± SD Haemoglobin (g/L)	129.4 ± 18.05	127.7 ± 18.5	0.459
High‐risk mechanism of injury *n* (%)	170 (66.2%)	419 (67.9%)	0.635
Trauma calls *n* (%)	38 (14.8%)	155 (25.1%)	0.001*
Trauma surgical involvement prior to scan	26 (10.1%)	30 (4.9%)	0.006
Clinical evaluation prior to scan			< 0.001*
Not examined	5 (1.95%)	10 (1.62%)	0.777
ED physicians	234 (91.1%)	597 (96.7%)	0.001*
Surgical team	18 (7%)	10 (1.62%)	< 0.001*

Abbreviations: bpm, beats per minute, Tachycardia HR ≥ 120 bpm; GCS, Glasgow Coma Scale; HR, heart rate; RR, respiratory rate, breaths per minute, tachypnoea defined as respiratory rate ≥ 25; SBP, systolic blood pressure, hypotension SBP ≤ 90mmHg; SpO_2_, oxygen saturation.

^a^ Factors impairing communication include a history of cognitive impairment (e.g., dementia), altered consciousness measured by reduced Glasgow Coma Score (GCS) < 14, intoxication or a non‐English speaking background.

^b^ Threshold values necessitating a hospital trauma call.

* *p* < 0.05.

Vital signs at triage (Table 1) were similar between both groups, including HR, SBP and GCS. Intervention patients had a small but statistically significant lower mean SpO2 (97% ± 2.6% vs. 96.1% ± 2.5%) and slightly higher RR (median (IQR) 17 (3) vs. 18 (4) breaths per min), both *p* < 0.001. Haemoglobin at presentation and proportion of patients who were tachycardic, hypotensive, tachypnoea or with GCS < 14 at triage were statistically similar between groups. While there was no statistically significant difference between the proportion of patients with high‐risk mechanisms of injury (Baseline 66.2% vs. 67.9%, *p* = 0.635), there was a significantly higher proportion of trauma calls in Intervention (14.8% vs. 25.1%, *p* = 0.001).

#### 3.1.1 Clinical Evaluation Prior to CT

Majority of patients were examined by an ED physician, with higher prevalence in Intervention (91.1% vs. 96.7%, *p* < 0.001). For Baseline, surgical team examination prior to CT (7% vs. 1.62%, *p* < 0.001), and surgical team involvement in decision making prior to scans (10.1% vs. 4.9%, *p* = 0.0006) was more common. A minority of patients had no documented examination prior to CT (1.95% vs. 1.62%, *p* = 0.77).

#### 3.1.2 Initial CT

There was a statistically significantly higher proportion of patients who underwent an initial WBCT scan in the Intervention group (Baseline 17.1% vs. Intervention 47.8%, *p* < 0.05). Although not statistically significant the median (IQR) for initial WBCT scan yield was lower for Intervention (Baseline 0.2(0.2) vs. 0(0.2), *p* = 0.21). For both groups, it was most common for patients who did not undergo WBCT to undergo selective CT scans involving two body regions (Baseline 40.38% vs. 59.62%, *p*‐value < 0.001). There was no difference in proportion of WBCT in patients who had at least one factor impairing communication in Intervention (impaired communication 46.1% vs. no factors impairing communication 53.9%, p = 0.7). However, there were statistically fewer WBCT scans in patients with impaired communication in Baseline compared to those without (34.1% vs. 65.9%, *p* = 0.02) (Table 2).

**TABLE 2:** Initial CT scan regions performed, and percentage of patients scanned (including those who underwent WBCT) with injury detected.

	Baseline		Intervention	
	Scanned *n* (%)	Injury detected *n* (%)	Scanned *n* (%)	Injury detected *n* (%)
Initial WBCT (%)	44 (17.1%)^a^		295 (47.8%)^a^	
Initial Selective CT B (%)	211 (99.1%)^a^	32 (12.6%)	306 (95%)^a^	78 (13%)
Initial Selective CT C spine (%)	210 (98.6%)^a^	12 (4.74%)	303 (94.1%)^a^	19 (3.2%)
Initial Selective CT chest (%)	37 (17.4%)	40 (46.5%)^a^	59 (18.3%)	113 (29.9%)^a^
Initial Selective CT abdomen (%)	2 (0.94%)	5 (10.2%)	11 (3.4%)	25 (7.6%)
Initial Selective CT pelvis (%)	2 (0.94%)	4 (8.2%)	11 (3.4%)	33 (10%)
Initial Selective CT other (%)	2 (0.94%)	1 (50%)	8 (2.48%)	4 (50%)
Initial WBCT yield median (IQR)	0.20 (0.20)		0 (0.20)	
Overall initial scan yield median (IQR)	0 (1)		0 (1)	
Odds ratio (of detecting injury with the initial scan) for intervention compared to baseline			0.876	

*Note:* Injury detected (%) refers the percentage of scans performed in that region inclusive of WBCT that were positive for traumatic injury in that region. Yield for injuries defined as one or more trauma‐related positive findings in a region/total number of regions scanned. Most common selective CT other performed was of the thoraco‐lumbar region.

Abbreviations: CTB, CT brain; CT‐Cspine, CT cervical spine.

^a^ *p* < 0.05 for between group comparisons.

In patients who did not undergo WBCT, CT‐B and CT C‐spine were the most common initial scans performed, with a statistically higher proportion of initial selective CT‐B (99.1%) and CT C‐spine (98.6%) for Baseline compared to intervention (CT‐B 95.0%; CT C‐spine 94.1%) groups, *p* = 0.013. There was no statistically significant difference in proportion of patients who underwent initial CT‐chest (17.4% vs. 18.3%), CT‐abdomen (0.9% vs. 3.4%) or CT pelvis (0.9% vs. 3.4%) across groups, all *p* > 0.05. Finally, the proportion of other CT scans was similar across both groups (0.94% vs. 2.48% *p* = 0.33).

Table 2 also presents prevalence of injuries detected by region, the median (IQR) yield of initial scans for injury across all regions was the same in both groups 0 (1).

The odds of detecting an injury during the initial scan across regions was 12.4% lower for Intervention compared to Baseline (Odds Ratio 0.876), however this difference was not statistically significant. There was no difference in the proportion of patients with injuries detected in both groups for head (12.6% vs. 13%), cervical spine (4.7% vs. 3.2%), abdomen (10.2% vs. 7.6%) and pelvis (8.2% vs. 10%) scans, all *p* > 0.05. There was a statistically significant lower prevalence of chest injuries on CT chest in Intervention (46.5% vs. 29.9%, *p* = 0.005).

#### 3.1.3 Injury Types

Bleeds were the most common type of head injury found on those who underwent CT‐B or WBCT for both groups (22/255, 8.63% vs. 69/601, 11.5%), noting that half in each group had serious intracranial bleeds such as subdural haematomas (Baseline 11/22, Intervention 34/69) while the other half were scalp haematomas (Baseline *n* = 11, Intervention *n* = 35) and subgaleal haematomas (Baseline and Intervention *n* = 4). The most common neck injuries were stable cervical spine fractures (Baseline 6/254, 2.36% vs. 12/598, 2.0%) and isolated injuries in CT‐chest were chest wall fractures (Baseline 25/81, 30.9% vs. 65/354, 18.36%), for which rib fractures were most common (Baseline 15/25, 60% vs. 54/65, 83%). Multiple chest injuries were also common (Baseline 8/81, 9.9% vs. 24/354, 6.78%) and typically involved rib fractures with associated pneumothorax or haemothorax. Organ lacerations were the most commonly found abdominal injury in Baseline (3/46, 6.5% vs. 0/306, 0%) compared to lumbar spine injuries in the Intervention (1/46, 2.2% vs. 18/306, 5.8%). In the pelvis, fractures of the femur (Baseline 2/46, 4.34% vs. 11/306, 3.59%) and pelvic ring (Baseline 1/46, 2.17% vs. 7/30, 2.28%) predominated. Pelvic haematomas (Baseline 1/46, 2.17% vs. 6/306, 1.96%) and sacral spine fractures (Baseline 0/46, 0% vs. Intervention 5/306, 1.63%) were also detected. Further detail regarding injuries is summarised in Table S2.

#### 3.1.4 Clinical Suspicion for Injury

There was no difference in clinical suspicion for injury in at least one body region between groups (94.5% vs. 92.1%, *p* = 0.250). When assessed by body region, there was a statistically higher proportion of patients in Intervention suspected of having chest injuries (28.8% vs. 37.1%) and abdominal injuries (7.4% vs. 15.1%), *p* < 0.05 for both. There was a statistically higher proportion of Baseline patients suspected of head (72% vs. 57.4%) and/or neck injuries compared to Intervention (62.3% vs. 51.5%), *p* < 0.01 for both. There was no difference in the proportion of patients with suspected pelvic injuries (Baseline 10.1% vs. 14.3%, *p* = 0.1). When there was clinically suspected injury in a region, there was no significant difference between both groups for injuries detected for most regions except chest. The number of injuries detected on CT chest was lower for Intervention (63.0% vs. 42.6%, *p* = 0.02) (Tables 3 and 4).

**TABLE 3:** Number and proportion of patients with documented clinical suspicion of injury by body region; number and proportion of those patients who were scanned and number with proportion of those patients with scan positive for injury.

Region	Clinical suspicion, *n* (%)			Patients scanned, *n* (%)			Positive scans, *n* (%)		
	Baseline	Intervention	*p*	Baseline	Intervention	*p*	Baseline	Intervention	*p*
Head	185 (72%)	354 (57.4%)	**< 0.001**	185 (100%)	353 (99.7%)	**< 0.001**	29 (15.7%)	66 (18.7%)	0.407
Cervical spine	160 (62.3%)	318 (51.5%)	**0.005**	160 (100%)	315 (99.1%)	**0.003**	10 (6.25%)	14 (4.4%)	0.507
Chest	74 (28.8%)	229 (37.1%)	**0.019**	54 (73%)	204 (89.1%)	**< 0.001**	34 (63%)	87 (42.6%)	**0.016**
Abdomen	19 (7.4%)	93 (15.1%)	**0.002**	16 (84.2%)	82 (88.2%)	**0.02**	2 (12.5%)	13 (15.9%)	1
Pelvis	26 (10.1%)	88 (14.3%)	0.1	9 (34.6%)	61 (69.3%)	**0.001**	3 (33.3%)	23 (37.7%)	1
All regions	243 (94.5%)	568 (92.1%)	0.25						

*Note:* Bold values represents *p* < 0.05 as significant.

**TABLE 4:** Number and proportion of patients with no documented clinical suspicion of injury by body region, number and proportion of those patients who were scanned and number of positive scans for injury.

Region	No documented clinical suspicion, *n* (%)			Patients scanned, *n* (%)			Positive scans, *n* (%)		
	Baseline	Intervention	*p*	Baseline	Intervention	*p*	Baseline	Intervention	*p*
Head	72 (28%)	263 (42.6%)	**< 0.001**	70 (97.2%)	248 (94.3%)	0.54	3 (4.3%)	12 (4.83%)	1
Cervical spine	97 (37.7%)	299 (48.5%)	**0.005**	94 (96.9%)	283 (94.6%)	0.584	2 (2.1%)	5 (1.77%)	0.684
Chest	183 (71.2%)	388 (62.9%)	**0.019**	27 (14.8%)	149 (38.4%)	**< 0.001**	6 (22.2%)	26 (17.4%)	0.586
Abdomen	238 (92.6%)	524 (84.9%)	**0.002**	30 (12.6%)	223 (42.6%)	**< 0.001**	3 (10%)	12 (5.38%)	0.4
Pelvis	231 (89.9%)	529 (85.7%)	0.1	37 (16%)	244 (46.1%)	**< 0.001**	1 (2.7%)	10 (4.1%)	1

*Note:* Bold values represents *p* < 0.05 as significant.

In patients without clinical suspicion for injury (Table 4), there was no difference in proportion of patients who underwent an initial CT‐B (Baseline 97.2% vs. 94.3%, *p* = 0.54) or initial CT‐C spine (96.9% vs. 94.6%, *p* = 0.58). However in the absence of documented clinical suspicion, intervention patients were more likely to undergo CT‐chest (14.8% vs. 38.4%), CT‐abdomen (12.6% vs. 42.6%) and CT‐pelvis (16.0% vs. 46.1%), *p* all < 0.001. When there was no documented clinical suspicion for injury, positive findings were similar between groups, with the highest injury prevalence in the chest (Baseline 15.0% vs. 22.0%).

#### 3.1.5 Incidental Findings

There was a statistically higher proportion of incidental findings requiring further assessment in Intervention (Baseline 39/257, 15.18% vs. 146/617, 23.66%, *p* = 0.005). This difference remained when compared by category, with a higher proportion of category 1 (Baseline 4/257, 1.56% vs. 30/617, 4.86%) and category 2 (Baseline 35/257, 13.62% vs. 116/617, 18.80%) incidental findings, *p* = 0.007. There was no difference in median (IQR) of number of regions with incidental findings between groups, 1(0), *p* = 0.3. The most common category 1 finding was lobar pneumonia, and category 2 finding was a thyroid nodule (Table S3).

#### 3.1.6 Radiation Exposure

Mean DLP was higher in Intervention (1580.5 ± 1420.4 mGy cm) compared to Baseline (1216 ± 468.4 mGy cm), *p* < 0.001.

#### 3.1.7 Patient Disposition From ED

There was a statistically significant difference in patient disposition between Baseline and Intervention (*p* < 0.001). More patients were discharged home in Baseline (39.30% vs. 30.31%, *p* = 0.01) and more patients were admitted for short stay observation in Intervention (21.72% vs. 14.01%, *p* = 0.01). There was no significant difference between groups for ward admissions (Baseline 44.4% vs. 46.5%), ICU (Baseline 0.78%, *n* = 2 vs. 1.3% *n* = 8) and transfers to operating theatre (Baseline 0.78%, *n* = 2 vs. 0.16%, *n* = 1), *p* > 0.05 all comparisons. Two deaths in the ED were found in Baseline, with 0 for Intervention, therefore this was not able to be statistically compared.

### 3.2 Radiology Impact

#### 3.2.1 Time to Scan

A longer time from presentation to initial scan was observed for Intervention (177.35 ± 119.68 min vs. 233.67 ± 150.37 min, *p* < 0.001). When comparing the time taken from presentation to a selective scan in comparison to a WBCT, there was no significant difference in times for Baseline (selective 172.88 ± 99.95 vs. 199.00 ± 188.25 min; *p* = 0.68). However, for Intervention the time from presentation to scanner was significantly longer if a WBCT was performed (255.95 ± 148.32 min) compared to selective CT (213.26 ± 149.55 min), *p* < 0.001 (Table 5).

**TABLE 5:** Impact on radiology workflow, including turnaround times for scanning and reporting of initial scans and prevalence, extent and yield of additional scans.

	Baseline	Intervention	*p*
Time from presentation to initial scan (mean ± SD, minutes)	177.35 ± 119.68	233.67 ± 150.37	< 0.001
Time taken from presentation to initial WBCT versus selective scan, (mean ± SD, minutess)	199.00 ± 188.25 versus 172.88 ± 99.95	255.95 ± 148.32 versus 213.26 ± 149.55	< 0.001
Time for radiology trainee report (mean ± SD, minutes)	25.16 ± 17.34	32.43 ± 25.28	< 0.001
Time for radiologist report (mean ± SD, minutes)	11.24 ± 14.91	13.95 ± 23.35	0.159
Total interpretation time (mean ± SD, minutes)	28.81 ± 22.40	39.37 ± 35.86	< 0.001
Total interpretation time by number of regions scanned (mean ± SD, minutes)	11.30 ± 8.75	11.83 ± 10.44	0.528
Additional scans *n* (%)	14 (5.45%)	77 (12.48%)	0.001
Additional scans when initial WBCT not performed *n* (%)	13 (6.1%)	61 (18.9%)	< 0.001
Additional regions scanned Median (IQR)	1 (1)	2 (2)	0.167
Time from presentation to additional scan (mean ± SD, minutes)	704.00 ± 513.03	849.68 ± 613.29	0.323

#### 3.2.2 Reporting Time

Mean trainee and overall reporting time increased from Baseline 25 and 28 min respectively, to Intervention 32 and 39 min, *p* < 0.001 both comparisons. There was no significant difference in consultant radiologist mean reporting time between groups (Baseline 11.24 ± 14.91 min vs. 13.95 ± 23.35 min, *p* = 0.159), or in overall reporting time (inclusive of radiologist and trainee) when indexed to the number of regions scanned (Baseline 11.30 ± 8.75 min vs. 11.83 ± 10.44 min, *p* = 0.52).

#### 3.2.3 Additional Scans

There was a significant increase in prevalence of additional scans in Intervention (5.45% vs. 12.48%), including those who underwent initial WBCT, *p* = 0.001. There was also a significant increase in additional selective CT if WBCT was not initially performed (Baseline 6.1% vs. 18.9%, *p* < 0.001). There was no significant difference in the median number of additional body regions scanned between Baseline (1 region) and Intervention (2 regions, *p* = 0.17). A higher additional CT scan yield for Baseline compared to Intervention did not reach statistical significance (Baseline median (IQR) 0(1) vs. Intervention 0(0); *p* > 0.09). The median (IQR) number of trips to scanner was 1(0) for both groups, however there was a greater overall number of trips required for Intervention (*p* = 0.002). There was no significant difference in the time to an additional CT scan from initial presentation between both groups (*p* = 0.32).

## 4 Discussion

With a dedicated TSU, our institution saw more trauma calls and an increased proportion of patients examined by an ED physician before CT scanning—positive findings in terms of ensuring appropriate trauma‐directed care. Reassuringly, the percentage of patients without a documented pre‐scan clinician examination was similarly low in both groups. Unexpectedly, surgical unit involvement before initial CT decreased. We postulate that scans during Intervention were being requested prior to surgical team involvement in anticipation of TSU subsequently requesting WBCT to rule out occult injuries.

In terms of CT coverage, during Intervention, more initial WBCTs were performed, with no significant difference in scan yield by region except for the chest, where fewer injuries were found. Presence of patient communication barriers did not impact the proportion of WBCT performed in Intervention but was associated with fewer WBCTs in Baseline. This could be due to the relative ease of obtaining collateral history pre‐COVID, while lingering visitation restrictions during Intervention might have made obtaining collateral history to rule out injuries more challenging. This reflects our findings where patient with impaired communication were more likely to have a WBCT in Intervention when compared to Baseline.

Selective scans in both groups were skewed towards CTB and CT‐C spine, possibly due to the Canadian head and C‐spine guidelines recommending imaging for > 65 year olds when head or neck injury cannot be excluded clinically [21]. The number of initial selective CT chest, abdomen, pelvis and CT other (commonly CT thoracolumbar spine) were similar across groups.

The injury rates we found in each region scanned were slightly lower than those reported in the 2021–2022 state trauma registry for all major trauma patients: 21% head injuries, 12% extremity or spine injuries, and 10% chest and abdominal injuries. This likely reflects our Level 2 trauma centre designation, with a relatively high prevalence of low‐impact trauma presentations (e.g., fall from standing height). Major trauma patients are generally preferentially directed to Level 1 trauma centres and are not common in our presenting population.

Intervention saw higher clinical suspicion for chest and abdominal injuries, while Baseline had higher suspicion for brain and cervical spine injuries. Where clinical suspicion of injury was present, injury prevalence across groups was similar except for lower chest injury findings in Intervention. Ho et al. reported clinical suspicion ascertained from physical exam combined with the presence of high‐risk factors (e.g., anticoagulation) can be highly sensitive (94%) for injuries beyond the head and neck region in populations like ours—geriatric blunt trauma [22]. However, this study found 1.6% of patients would be missed using this tool, similar to our patients with no clinical suspicion for injury.

In our study, in the absence of clinical suspicion, the percentage of positive scans was low across both groups for all scans, with the highest being for CT‐chest. Regardless of clinical suspicion of chest injury, the highest injury prevalence was in the chest region for both Baseline and Intervention. The prevalence of abdominal and pelvic injuries detected was low. The majority were musculoskeletal (fracture), suggesting plain film, non‐contrast spine or pelvic‐ring directed CT may be sufficient for low‐energy trauma based on level of clinical concern. The higher injury prevalence on scans with a clinical suspicion for injury reflects well upon clinical acumen. Positive findings in the low clinical suspicion subgroup highlights the role of imaging in detecting occult injuries which Roberts et al. report a rate of 25%, particularly in the setting of distracting injuries alongside other factors (e.g., frailty, mechanism of injury) [23].

However, it is notable that a recent meta‐analysis found routine WBCT for trauma neither improved mortality nor reduced overall hospital length of stay [24]. There is increasing inter‐speciality recognition of the importance of employing selective CT for use of finite resources [21, 25]. Balancing CT use with the risk of missing injuries is challenging, but validated clinical decision tools including NEXUS CT head, cervical spine, and chest aid in decision‐making [21].

A consequence of greater scan coverage in Intervention was greater radiation exposure and more incidental findings necessitating follow up. More patients in Intervention were admitted to the short‐stay unit rather than discharged home, possibly for a longer monitoring period prior to discharge, further investigations, or to await results (e.g., imaging reports). Category 1 incidental findings might also necessitate further inpatient hospital team involvement.

Regarding impacts to Radiology services, the intervention group experienced longer scan turnaround and reporting times, along with increased additional scan requests, even if WBCT was performed initially. Contributing factors include expanded coverage and complexity of scans in Intervention period (e.g., more chest, abdomen, and pelvic CT scans performed, often with intravenous contrast, for trauma). The intervention period also coincided with an increase in major trauma ED presentations, as identified by state registry, possibly contributing to an increase in ED presentation and CT wait times [26]. However, no significant difference was found in reporting times when indexed by number of regions scanned, indicating that increased coverage is the major contributor to the overall increased reporting time. Although our study did not investigate this, it is possible as reported by Balint et al. that the increase in regions scanned and the associated workload (e.g., phone calls to physicians) can have indirect effects on the accuracy of radiology trainee reports [27].

Although the greater number of additional scans in Intervention affected Radiology services, the higher injury prevalence found on additional scans in Baseline was not statistically significant, likely due to insufficient statistical power. These results could also reflect a surgical preference for “completion scans” to ensure no injuries are missed. Kelleher et al. similarly found completion CT chest, abdomen and pelvis scans rarely unveiled acute injuries in patients with low‐velocity trauma who had acute head and/or C‐spine injuries [28]. Completion scans may play a bigger role when physical examination is less sensitive, e.g. in the abdominopelvic region [29].

This study has several limitations. This was a retrospective study utilising written electronic medical record documentation, which required some subjective interpretation by data collectors, e.g. presence of clinical suspicion. As the study was performed at a Level 2 trauma centre on patients who had undergone CT‐C spine and/or additional regions for trauma, as identified for inclusion via the RIS, results are not generalisable to patients not identified by these means, nor to the severity of injury more common in a Level 1 trauma centre or the population captured in the REACT‐2 trial. We did not collect data on the use of other modalities (e.g., point of care ultrasound or plain radiograph), extent of examination performed, level of experience of assessing ED physicians or surgical registrars, difference in levels of experience of surgical registrars pre and post‐TSU, presence or absence of collateral history and distracting injuries, which could influence the decision for use of CT. We did not comprehensively assess for missed findings with selective CT vs. WBCT, as our primary aim was to assess CT utilisation. Although additional CTs may be considered a surrogate for missed findings, referrals also depended on clinical suspicion, similar to initial CT referrals. Despite more WBCT in Intervention, additional scans also statistically significantly increased, suggesting a shift in practice towards comprehensive CT coverage. During Intervention, the TSU was primarily responsible for trauma referrals, with other surgical units involved after hours. Other confounding factors to consider include staff changes and ED demand variations between study periods. ED disposition data was used as a surrogate for acute patient outcomes. The significance of injuries identified in terms of patient outcome was not studied, with one prior study indicating that radiographically occult rib fractures do not adversely impact patient outcome [30]. This was beyond the scope of the present work but is a vital topic for future study, impacting the utilisation of finite healthcare resources.

## 5 Conclusion

Introducing a dedicated trauma service at a Level 2 trauma centre led to more CT scans overall, with no significant increase in scan yield and low injury prevalence in a predominantly geriatric population. The majority of patients were assessed by an ED physician, increased compared to Baseline, however received greater ionising radiation and with more incidental findings identified. A larger proportion of trauma patients remained longer in the hospital for observation. Time to CT scan, reporting, and additional CT scans increased. These findings have led to a multidisciplinary discussion between ED, TSU, and Radiology regarding appropriate resource utilisation at our institution. Proposed changes to our organisation's trauma guidelines include reserving WBCT for high‐energy trauma or clinically suspected visceral injuries. For the majority of our institution's trauma population, patients > 65 years old, if there is suspected chest, abdominal or pelvic trauma with a low clinical suspicion of visceral injury, non‐contrast chest and/or targeted AP imaging of spine and pelvis is suggested as the default approach to identify musculoskeletal injuries and minimise incidental findings. Our study highlights the need for appropriately targeted patient care and judicious use of limited imaging resources.

## Conflicts of Interest

The authors declare no conflicts of interest.

## Supporting information


Data S1.

## Data Availability

The data that support the findings of this study are available from the corresponding author upon reasonable request.
