# Supplementary material for: CT Utilisation in Emergency Department (ED) Assessment of Patients With Suspected Polytrauma: Impact of a Dedicated Trauma Surgical Team
Source: J Med Imaging Radiat Oncol. 2025 Feb 22;69(3):317–27. doi: 10.1111/1754-9485.13843 (PMC12120588; doi:10.1111/1754-9485.13843)
Supplement: Supplementary file 1 — Data S1. [file ARA-69-317-s001.docx]

**Supplementary Tables**

**Table 1.** CT Trauma parameters. Please note these are representative values only, for a typical approximately 80kg male patient, with chest, abdomen and pelvis parameters in particular dependent upon patient body habitus and compliance with the examination.

| **Parameters** | **Brain** | **C Spine** | **Chest** | **Abdomen and Pelvis** |
| --- | --- | --- | --- | --- |
| **Scan Mode** | Helical | Helical | Helical | Helical |
| **Collimation (mm)** | 19.2 | 38.4 | 57.6 | 57.6 |
| **Recon thickness ST (mm)** | 3 | 3 | 3 | 3 |
| **Recon Thickness Bone (mm)** | 0.6 | 0.6, 2 | 0.6, 2 | 0.6, 2 |
| **Rotation time** | 1 | 1 | 0.25 | 0.5 |
| **Pitch** | 0.55 | 0.8 | 0.6 | 0.6 |
| **kVp** | 120 | 120 | 100 | 100 |
| **Tube current (mA)** | 104 - 214 | 94 - 387 | 200 - 700 | 180 - 230 |
| **Contrast type** | - | - | Iohexol (Omnipaque 350, GE Healthcare) | |
| **Contrast rate (ml/sec)** | - | - | 2.5 | |
| **Contrast volume (ml)** | - | - | 90 | |
| **Contrast delay (s)** | - | - | 20 | 70 |

**Table 2.** Most common types of injuries in each body region in baseline compared to intervention group

|  | **Baseline** |  | **Intervention** | |
| --- | --- | --- | --- | --- |
| **Head** | **n** | **% scanned** | **n** | **% scanned** |
| Fractures | 3 | 1.18 | 4 | 0.66 |
| Bleeds (Total)  Intracranial haematoma  Isolated Scalp haematoma  Isolated Subgaleal | 22  11/22 (50%)  11/22 (50%) 4/22 | 8.63  4.32  4.32  1.56% | 69  34/69 (49.3%)  35/69 (50.7%) 4/69 | 11.48  5.66  5.82  0.66 |
| Contusions | 3 | 1.18 | 2 | 0.333 |
| Multiple injuries | 3 | 1.18 | 3 | 0.499 |
| Other injuries | 1 | 0.39 | 0 | 0 |
| Total scanned | 255 |  | 601 |  |
| **Cervical spine** | **n** | **% scanned** | **n** | **% scanned** |
| Stable C spine fracture | 6 | 2.36 | 12 | 2.00 |
| Unstable C spine fractures | 4 | 1.57 | 3 | 0.502 |
| Malalignment | 1 | 0.394 | 1 | 0.167 |
| Other | 1 | 0.394 | 1 | 0.167 |
| Total scanned | 254 |  | 598 |  |
|  |  |  |  |  |
| **Chest** | **n** | **% scanned** | **n** | **% scanned** |
| Chest wall fractures  Isolated Rib fractures | 25  15/25 | 30.9 | 65  54/65 | 18.4 |
| Bleeds | 1 | 1.23 | 1 | 0.282 |
| Thoracic spine fractures | 4 | 4.94 | 14 | 3.955 |
| Lung contusions | 1 | 1.23 | 2 | 0.565 |
| Pneumothorax | 0 | 0 | 1 | 0.282 |
| Multiple injuries | 8 | 9.88 | 24 | 6.77 |
| Chest adjacent injuries | 1 | 1.23 | 6 | 1.70 |
| Total scanned | 81 |  | 354 |  |
|  |  |  |  |  |
| **Abdomen** | **n** | **% scanned** | **n** | **% scanned** |
| Laceration | 3 | 6.52 | 0 | 0 |
| Contusions | 1 | 2.17 | 1 | 0.327 |
| Bleeding | 0 | 0 | 6 | 1.961 |
| Lumbar spine fractures | 1 | 2.17 | 18 | 5.88 |
| Total scanned | 46 |  | 306 |  |
|  |  |  |  |  |
| **Pelvis** | **n** | **% scanned** | **n** | **% scanned** |
| Pelvic fractures | 1 | 2.17 | 7 | 2.29 |
| Femoral fractures | 2 | 4.348 | 11 | 3.59 |
| Sacral spine fracture | 0 | 0 | 5 | 1.63 |
| Haematoma | 1 | 2.17 | 6 | 1.96 |
| Multiple injuries | 0 | 0 | 4 | 1.31 |
| **Total scanned** | **46** |  | **306** |  |

Head injuries: fractures included fractures of calvarium, skull base, facial bones. Bleeds included subdural, subarachnoid, intraventricular, extradural and subgaleal bleeds. Multiple injuries were a combination of injuries such as fracture and associated bleed. Other injuries included soft tissue injury. We attempted to collect data on presence of vascular injuries such as active bleeds, pseudoaneurysm, intracerebral dissections, arteriovenous malformations and transection but none of our patients had these findings.

Neck injuries: fractures were recorded as either stable or unstable c-spine fractures. We attempted to collect data on any perforations, contusions, cervical spine haematoma, vascular injuries (eg. Pseudoaneurysm, intimal tears, transections, arteriovenous fistula), spinal cord injury and injury in adjacent body regions of scan such as thoracic spine fractures, but none of our patients had these findings.

Chest injuries were recorded as chest wall fractures (inclusive of rib fractures, sternal, scapular or clavicular fractures), thoracic spine fractures, bleeds (inclusive of chest wall, haemothorax, mediastinal, haemopericardium), lung contusions, pneumothorax and chest adjacent injuries for example humeral fracture. We also looked for presence of lung lacerations, pneumomediastinum, pneumopericardium and thoracic spinal cord injury but no patients had this finding.

Under category of abdominal injuries, the following if present were recorded: lacerations, contusions, non-organ haematomas (inclusive of retroperitoneal, hemoperitoneum, extraperitoneal), vascular injuries (active bleeds, pseudoaneurysm, intimal injury, transections) and lumbar spine fractures. There were no injury in adjacent regions to abdomen nor lumbar spinal cord injuries.

Pelvic injuries include fractures of pelvic ring, femur or sacral spine, haematomas or multiple injuries. None of our patients had pelvic organ contusions, lacerations, vascular injuries or spinal cord/cauda equina injuries.

**Table 3.** Incidental Findings

|  | **Baseline** | | **Intervention** | |
| --- | --- | --- | --- | --- |
| Presence * | 39 (15.18%) | | 146 (23.66%) | |
| Category 1 ** | 4 (1.56%) | | 30 (4.86%) | |
| Category 2 ** | 35 (13.62%) | | 116 (18.80%) | |
|  |  | |  | |
| **HEAD** | **Cat 1** | **Cat 2** | **Cat 1** | **Cat 2** |
| Suspected normal pressure hydrocephalus |  |  |  | 4 |
| Infection |  |  | 2 |  |
| Infarct |  |  | 1 |  |
| Intracranial aneurysm |  |  | 1 |  |
| Lucent bone lesion |  | 2 |  | 1 |
| Tumour |  |  |  | 2 |
| Colloid cyst |  |  |  | 2 |
| Vascular lesion |  | 1 |  |  |
| Scalp lesion |  |  |  | 1 |
| Massive ventriculomegaly |  |  | 1 |  |
|  |  |  |  |  |
| **NECK** |  |  |  |  |
| Thyroid nodule / goitre |  | 10 |  | 14 |
| Cervical lymphadenopathy |  | 1 |  | 2 |
| Parotid mass |  |  |  | 2 |
|  |  |  |  |  |
| **CHEST** |  |  |  |  |
| Lobar pneumonia / aspiration |  |  | 9 |  |
| Pulmonary embolism | 1 |  | 3 |  |
| Pulmonary oedema | 2 |  | 2 |  |
| Pleural effusion | 1 |  | 2 |  |
| Pulmonary nodule |  | 10 |  | 16 |
| Non-specific inflammation or infection |  | 1 |  | 6 |
| Chronic lung disease |  |  |  | 6 |
| Other |  | 1 |  | 5 |
|  |  |  |  |  |
| **ABDOMEN/PELVIS** |  |  |  |  |
| Abdominal aortic aneurysm <5mm |  | 2 |  | 8 |
| Portal vein thrombosis |  |  | 1 |  |
| GI tract inflammation |  |  | 4 |  |
| GU tract inflammation |  |  | 1 |  |
| Benign appearing solid visceral lesion |  | 6 |  | 14 |
| Suspicious appearing mass for primary malignancy or metastasis |  |  |  | 14 |
| Lymphadenopathy or peritoneal nodularity |  |  |  | 2 |
| Gallstones or kidney stones |  |  |  | 3 |
| Other |  | 1 |  | 12 |
|  |  |  |  |  |
| **SPINE** |  |  |  |  |
| Cord compression (secondary to metastasis) |  |  | 1 |  |
| Spondylodiscitis (probably chronic) |  |  | 1 |  |
| Lucent or sclerotic bone lesions |  |  |  | 3 |
| Nerve sheath tumour |  | 1 |  |  |
|  |  |  |  |  |
| **Extremities** |  |  |  |  |
| Femoral AVN |  |  | 1 |  |

* P-value for the presence of incidental findings between baseline and intervention groups, P=0.005. **P-value for category 1 and 2 findings in baseline and intervention groups, P=0.007. Statistical comparison between groups was performed at a patient level. For patients with multiple category 1 or 2 findings these results are represented in the table under the relevant region(s).

Other findings of the chest include breast mass, thick-walled oesophagus, and thoracic lymphadenopathy.

Benign appearing solid visceral lesions includes lesions of the liver (e.g. suspected haemangioma, complex hepatic cyst), pancreas (i.e. IPMN), adrenal/kidney (adenoma, AML), adnexa, uterus and endometrium

Other findings of the abdomen include liver cirrhosis, abdominal hernia, hydronephrosis without cause, partially imaged left upper quadrant cystic lesion, internal iliac artery aneurysm, gastric antrum thickening, endometrial thickening in post-menopausal women, ovarian teratoma, and gallbladder polyp.
